# Propolis and Diet Rich in Polyphenols as Cariostatic Agents Reducing Accumulation of Dental Plaque

**DOI:** 10.3390/molecules27010271

**Published:** 2022-01-02

**Authors:** Anna Kurek-Górecka, Karolina Walczyńska-Dragon, Rafael Felitti, Stefan Baron, Paweł Olczyk

**Affiliations:** 1 Department of Community Pharmacy, Faculty of Pharmaceutical Sciences in Sosnowiec, Medical University of Silesia in Katowice, 3 Kasztanowa St, 41-200 Sosnowiec, Poland; polczyk@sum.edu.pl; 2 Department of Temporomandibular Disorders, Medical University of Silesia in Katowice, 2 Traugutta Sq, 41-800 Zabrze, Poland; karolina.dragon@sum.edu.pl (K.W.-D.); sbaron@sum.edu.pl (S.B.); 3 Oral Rehabilitation and Prosthodontics, Private Practice, Felix Olmedo 3716, Montevideo 11700, Uruguay; rafael.felitti@gmail.com

**Keywords:** dental plaque, diet, propolis, polyphenols, SARS-CoV-2 infection

## Abstract

Conducted studies indicate the relationship between oral health and systemic diseases. Moreover, the latest research indicated that cariogenic bacteria may severely influence the course of SARS-CoV-2 infection and increase risk of COVID-19 complications. This article aims to review various applications of propolis and pay attention to a healthy diet rich in polyphenols, which may allow the reduction of dental plaque accumulation. A literature review has been conducted from June until November 2021. It showed that propolis could be a useful agent in decreasing the accumulation of dental plaque. Moreover, a diet rich in polyphenols prevents cariogenic bacteria and reduces the accumulation of dental plaque. A reduction of a dental plaque may influence the risk of a severe course of COVID-19. Therefore, propolis and a diet rich in polyphenols may play an important role in prophylaxis of systemic diseases. Recently, it has been proven that oral infection may affect cardiovascular system, musculoskeletal system, respiratory system, nervous system, as well as may be a risk factor for diabetes mellitus. These aspects should stimulate clinicians to further research about polyphenols.

## 1. Introduction

Dental plaque is the community of microorganisms found on a tooth surface (or on other hard surfaces inside the oral cavity, like dentures), as a biofilm, embedded in a matrix of polymers of both host and bacterial origin [1]. Microorganisms attach to other microorganisms in a way that allows them to survive and resist host defense mechanisms or antibiotic treatment. Biofilm, if not removed regularly, undergoes maturation, connected with a progressive shift from Gram-positive to Gram-negative anaerobic species, which results in biofilm formation under the gingival surface, where bacteria are allowed to grow profusely. It causes the progressive destruction of the tooth-supporting apparatus, which leads to periodontitis [2]. Periodontal pathogens and their products, including inflammatory mediators such as IL-6, could enter the bloodstream and influence distant organs. Chronic gingivitis and periodontitis has been found to be a risk factor for diabetes mellitus, cardiovascular disease, respiratory disease, rheumatoid arthritis, and recently, constituting a high risk for developing severe illness due to SARS-CoV-2 infection [3,4]. Gingivitis is a reactive condition that is reversible upon the improvement of oral hygiene. Periodontitis takes place when the periodontal condition has progressed beyond gingivitis into a chronic, destructive, irreversible inflammatory disease state. There are many ways of improving the reduction of dental plaque formation, one of them is using propolis and diet rich in polyphenols [5,6]. Propolis or bee glue is a resinous material produced by bees, well known for its therapeutic properties. Propolis may have a promising role in oral health in dentistry, but also as a natural supplement and functional food. The main propolis components are polyphenols being responsible for a beneficial impact on the oral microflora as well as antibacterial and anti-inflammatory action. Among the active components responsible for the antibacterial action are: galangin, chrysin, pinobanksin, quercetin, naringenin, apigenin, tt-farnesol, artepillin C, and phenolic acids as well as ursolic acid and bacarin [7,8,9,10,11]. Caffeic acid phenetyl ester (CAPE) and galangin constitute the major components responsible for anti-inflammatory action [12]. Therefore, mainly polyphenols display an important role in prevention of different diseases and a dental plaque accumulation. Due to their impact on pathogenic microorganisms and anti-inflammatory mode of action, they are promising agents in prevention of some systemic diseases and recently also SARS-CoV-2-infection [13]. 

## 2. Materials and Methods

A literature review was performed to search for the association between dental plaque, diet rich in polyphenols, and propolis as cariostatic agents. A broad search was conducted on the electronic databases Medline, PubMed, and Cochrane. The databases were searched from 1st June until 15th November, 2021. The search was filtered to include only papers published from 2015 to 2021, in both the Polish and English language. The following keywords were used in search to find proper articles: “propolis AND therapeutic use” OR “dental plaque AND complications” OR “dental plaque AND diet” OR “dental plaque AND prevention” OR “diet rich in polyphenols” (according to Medical Subject Headings). 

A preselection of the articles was made by reviewing their title and/or abstract, and those that did not meet the inclusion criteria were rejected. The following articles were included from the literature review:

- observational studies evaluating the association between dental plaque and systemic diseases
- randomized clinical trials (RCTs) and observational studies evaluating the impact of propolis on hygiene of oral cavity
- randomized clinical trials (RCTs) and reviews presenting the impact of food rich in polyphenols in prophylaxis of dental plaque accumulation

The following articles were excluded from the literature review:

- case reports
- articles published in other languages than Polish and English.

Two researchers then conducted an independent in-depth analysis of the remaining articles. During the formulation of the search strategy we asked three questions:

1. Is there an association between dental plaque and systemic diseases?
2. Can propolis play a role as a cariostatic agent?
3. What is the impact of diet containing polyphenols on oral cavity microbiome?

The quality of the included studies was assessed based on the adequacy of the study design to the research objective, risk of bias, reliability of results, statistical work, and quality of reporting. 

## 3. The Results

**Figure 1:**
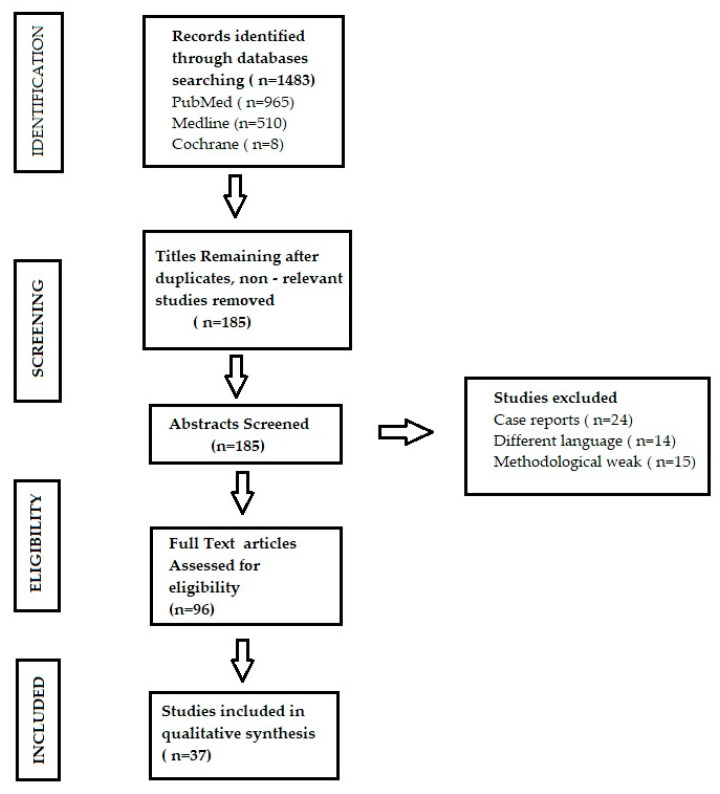
Flowchart for the review.

A total of 965 articles were found in the PubMed database, 510 articles in Medline database, and 8 articles in Cochrane database. After applying the inclusion and exclusion criteria and analyzing the abstracts, 37 articles were finally selected (Figure 1).

### 3.1. The Role of Dental Plaque in the Course of Systemic Diseases and SARS-CoV-2 Infection

**Figure 2:**
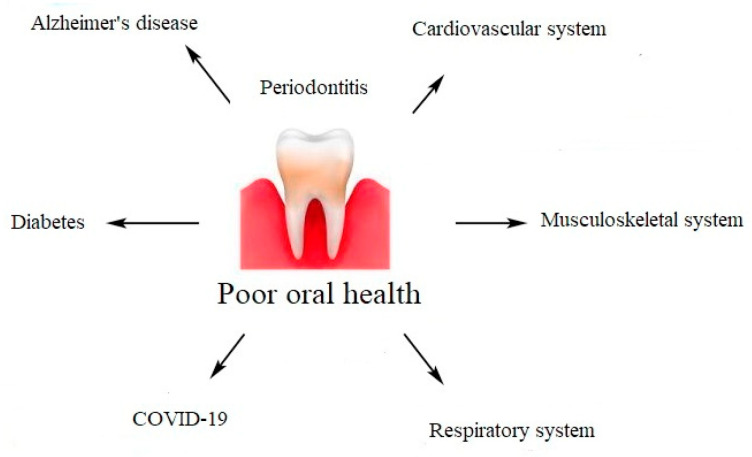
Impact of poor oral health on systemic diseases.

Scientific evidence has demonstrated that periodontal diseases are not just simple bacterial infections, but rather complex diseases of multifactorial complexity. The connection between the subgingival microbes, the host immune, and inflammatory responses seems to be very clear [14]. The mechanisms explaining the association between chronic periodontal disease and systemic diseases include a direct and an indirect route. The direct route results in ulceration in the lining of periodontal pockets, which can become a passage for bacteria into the systemic circulation. It leads to bacteremia, which allows bacteria to settle in distant organs aggravating existing disease conditions. The indirect route is based on the fact that chronic periodontal disease, being a significant source of inflammation, may play a role in other disease conditions in which inflammation is a major component [15]. A chronic oral infection such as periodontitis is a constant potential source of infection and has been considered as a risk factor for cardiovascular diseases, cerebrovascular diseases, peripheral arterial disease, respiratory diseases, and neurodegenerative diseases [16]. In addition, periodontitis has been described as a potential risk for increased morbidity and mortality during the course of diabetes, insulin resistance, rheumatoid arthritis, obesity, osteoporosis, Alzheimer’s disease, and COVID-19 (Figure 2). Evidence suggests a bi-directional relationship between periodontitis and systemic diseases. Periodontal diseases are of great importance in the course of many systemic diseases [17].

#### 3.1.1. Cardiovascular System

Cardiovascular diseases (CVD) are defined as disorders of the heart and blood vessels. An underlying cause of CVD is atherosclerosis. Atherosclerosis is a chronic, vascular inflammatory condition that results in lipids deposition in the arterial wall. The periodontal infections and cardiovascular diseases are related to each other. Many studies have shown that periodontitis usually results in higher systemic levels of C-reactive protein, interleukin (IL)-6, and neutrophils. Increasing inflammatory activity in atherosclerotic lesions, which potentially increases the risk for cardiac or cerebrovascular events may be the result of elevated inflammatory factors. Inflammation as a result of periodontitis increases systemic inflammation and oxidative stress, which contributes to and increases the already chronic inflammation present and in this way contributes to atherosclerosis and CVD [18,19,20]. It has been proven that some bacteria isolated from oral cavity may be associated with platelet aggregation, as far as bacterial infections may also contribute to the acute thromboembolic events. Oral bacteria involved in periodontal disease can infect blood vessels or in some other way promote plaque formation and, thus, CVD [21]. 

#### 3.1.2. Musculoskeletal System

Chronic periodontitis is associated with a higher risk of suffering from rheumatoid arthritis. The interrelationship between systemic osteoporosis, oral bone loss, tooth loss, and risk factors for these conditions has been observed, because a remarkable similarity in the pathogenesis of periodontal diseases and rheumatoid arthritis exists. In addition, periodontal disease is thought to be an initiative factor of the autoimmune inflammatory response, which is highly connected with rheumatoid arthritis [22,23,24].

#### 3.1.3. Respiratory System and SARS-CoV-2 Infection

Poor oral hygiene and periodontitis influence the incidence of pulmonary infections. The link between dental plaque and SARS-CoV-2 infection is based on direct and indirect mechanisms. The direct mechanism is related with angiotensin-converting enzyme II (ACE-2) receptors and is associated with greater expression of ACE-2 receptors by the altered oral microbiome [25]. It is known that ACE-2 receptors play a key role in SARS-CoV-2 entry into host cells. Therefore, a greater expression of ACE-2 receptors promotes SARS-CoV-2 infection on the epithelial cells of oral mucosa. Moreover, a colonization of dental plaque by respiratory pathogens could lead to aspiration of oral bacteria capable of causing pneumonia into the lungs. Other known mechanisms in periodontitis include alteration of the mucus surface by salivary enzymes, destruction of salivary pellicles by periodontal disease-associated enzymes and alteration of respiratory epithelium by cytokines from periodontal disease, also facilitating the infection of the epithelium by respiratory pathogens [20,26,27]. The oral cavity constitutes a reservoir for respiratory pathogens, therefore patients with dental plaque or periodontitis may be more likely to develop severe pneumonia [28,29,30]. The second, indirect mechanism is related to inflammatory pathways and bacterial superinfections [29]. The greater synthesis of cytokines and chemokines appears in the gingiva, which lead to increased levels of proinflammatory cytokines in the patient’s serum. Cytokines may alter the respiratory epithelium and lead to infection by respiratory pathogens such as SARS-CoV-2. Virus activates an immune response causing a “cytokine storm” [31]. Due to cytokine release syndrome, the hypercytokinemia is responsible for complications such as lung injury, hypercoagulation, multiorgan failure, and shock, during COVID-19. The inflammatory pathways that are involved in conditions such as diabetes, hypertension, obesity, or cardiovascular disease are the same that we observe in periodontal diseases. It is suggested, that there is a strong association between main comorbidities and increased risk of complications and death from COVID-19, and it may be connected with altered oral biofilms and periodontal disease. Moreover, indirect mechanism is based on bacterial superinfections. It is clear that altered microbiom lead to aspiration of microorganisms associated with oral diseases into lower respiratory tract and cause respiratory infections and potentially post-viral bacterial complications [13,32].

#### 3.1.4. Diabetes

Studies have shown that periodontal disease may be a risk factor for diabetes, but it is suggested that the relationship between diabetes and periodontal disease runs both ways: chronic infection and inflammatory response [21]. Severe periodontal disease can increase glucose levels, contributing to increased periods of time when the body functions with hyperglycemia. This puts people with diabetes at increased risk for diabetic complications. Pancreas cells responsible for insulin production can be damaged or destroyed by the chronic high levels of cytokines. It may induce Type 2 diabetes, even in otherwise healthy individuals with no other risk factors for diabetes. Therefore, periodontal diseases are responsible for aggravate insulin resistance and affect glycemic control [33,34,35,36,37,38]. 

#### 3.1.5. Alzheimer’s Disease

Recent comprehensive oral-health studies demonstrate the relationship between oral pathogen, inflammation, and Alzheimer’s disease (AD). In response to oral bacterial infection, pro-inflammatory cytokines are produced by the host [39]. Therefore, the increased level of cytokines lead to inflammation and may contribute to the brain inflammation that occurs among patients with Alzheimer’s disease. Moreover, dental plaque leads to periodontal diseases and changed microbiome. Periodontal pathogens such as *Porphyromonas gingivalis* and *Treponema dentricola* produce lipopolysaccharide (LPS). LPS constitutes a virulence factor and plays an important role in brain inflammatory process. Therefore, inflammation is a major factor responsible for neurodegeneration among patients [40]. Besides LPS, also gingipains are released by *Porphyromonas* *gingivalis*. Gingipains are classified as collagenases and trypsin-like cysteine proteinases and they are secreted by all strains of *Porphyromonas gingivalis*. Gingipains together with LPS can proteolytically activate kinases such as glycogen synthase kinase-3 β (GSK-3β), which phosphorylates neuronal tau protein [41]. Phosphorylated tau is an important agent, especially since the intraneuronal cytoskeletal alterations precede the formation of amyloid in patients with AD [42]. Additionally, periodontal pathogens such as *Treponema dentricola* and *Chlamydia pneumonia* were detected in postmortem brains derived from patients with Alzheimer’s disease. Moreover, *Porphyromonas gingivalis* is responsible for the peripheral and cerebral immune responses. Therefore, researchers put forward a hypothesis, that pathogens from oral cavity may invade the brain by crossing the brain–blood barrier. Periodontal pathogens may be associated with symptoms of Alzheimer’s disease [34].

### 3.2. Propolis as a Therapeutic Agent in Dentistry

Propolis consists of resinous substances collected by bees. The composition of propolis depends on its different origin. Based on morphology, behavior, and biological geography, there are 25 subspecies belonging to three or four major groups. The most common propolis is a poplar type, collected from Europe, North America, non-tropical regions of Asia, New Zealand, and even Africa [43]. However, generally it is mainly composed of resin and balsams (50%), it also includes essential oils (10%), wax (30%), and pollen (5%). Propolis is rich in bioactive substances such as: phenolic acids, flavonoids, amino acids, minerals, and vitamins. Phenolic compounds including phenolic acids and esters as well as flavonoids are the most numerous group of propolis components with respect to the quantity and type [44,45]. The characteristic components in temperate region propolis are flavonoids such as chrysin, galangin, pinocembrin, pinobanksin. Caffeic acid phenethyl ester (CAPE) is a characteristic constituent of temperate region propolis, while prenylated phenylpropanoids, for example, artepillin C and diterpenes constitute main components in propolis from tropical region propolis, especially Brazilian green propolis. The most important of phenolic acids are the following compounds: benzoic acid, cinnamic acid, caffeic acid, and ferulic acid. Among flavonoids, which are present in the propolis there are: flavones (chrysin, apigenin, acacetin), flavonols (galangin, kaempferol, quercetin), and flavanones (pinostrobin and pinocembrin). Among amino acids, there are: alanine, arginine, asparagine, aspartic acid, cystine, glutamic acid, glycine, histidine, hydroxyproline, isoleucine, leucine, lysine, methionine, proline, serine, threonine, tryptophane, tyrosine, valine [46,47,48]. Moreover, propolis contains minerals such as magnesium, calcium, potassium, sodium, cooper, zinc, manganese, iron, and vitamins such as B1, B2, B6, C, E. However, phenolic compounds influence beneficial properties of propolis regarding the oral cavity and teeth. As propolis possesses bacteriostatic, bactericidal, and anti-adherent properties against microorganisms, it is useful in reducing a dental plaque [12,49]. 

Moreover, propolis strongly inhibits bacterial glycosyltransferase so it is effective in the reduction of dental plaque [50]. 

Microbial dental plaque causes an acute and chronic inflammation of tissues surrounding the teeth and in that way leads to initiation of periodontal disease. Therefore, periodontal disease constitutes a predisposition to bacterial infections. In the light of the latest studies, a dental biofilm promotes bacterial infection and is connected with severe course of SARS-CoV-2 infection with its complications [13,51]. 

The antibacterial activity of propolis is connected with the direct action towards the microorganisms, and with indirect action towards the stimulation of the body immune system. 

The mechanisms of propolis action are based on its effect on the permeability of the cellular membrane of the microorganism, involving also the membrane disruption as well as bacterial cell wall and cytoplasm destroying. Moreover, propolis inhibits bacterial protein synthesis [52]. 

Antibacterial action of propolis is connected with the presence of flavonoids such as galangin, chrysin, pinobanksin, quercetin, naringenin, apigenin, tt-farnesol as well as phenolic acids like caffeic acid, benzoic acid, gallic acid, and cinnamic acid and bacarin or ursolic acid (Table 1) [7,8,9,10]. A study conducted by Lisbona-Gonzales et al. confirmed that the ethanolic extract of Spanish Propolis reduced bacterial counts of *Tannerella forsythensis* and *Porphyromonas gingivalis* [11].

Moreover, artepillin C (3,5-diprenyl-*p*-coumaric acid) is a phenolic compound present in Brazilian green propolis, which exhibits an antimicrobial effect [46]. Antimicrobial activity of propolis against *Streptococcus mutans* and *Lactobacillus* spp. has been confirmed by Dziedzic et al., and Elbaz and Elsayad. The mentioned strains turned out to be responsible for the biofilm formation [11,53]. 

Besides inhibiting oral pathogens growth, propolis decreases *Streptococci* adhesion to the teeth. The mentioned bacteria are known to be responsible for the formation of dental plaque [11]. According to Koo at al., Brazilian ethanol propolis extracts led to the reduction of the mentioned adherence of *Streptococcus mutans* and *Streptococcus sobrinus* as well as *Streptococcus cricetus.* The adherence of *Streptococcus mutans* and *Streptococcus sobrinus* cells and water insoluble glucan synthesis were markedly inhibited by all ethanol extracts of propolis from Brazil, showing significant reduction at all concentrations compared with the control (80% ethanol). Moreover, it was confirmed that ethanol extract of propolis from Northeastern Brazil inhibited the cell adherence and water insoluble glucan synthesis at lowest concentrations as 12.5 and 7.8 microg/mL, respectively. This extract showed the highest ability regarding an antiadherent action [54]. 

Another study conducted by Duarte et al. demonstrated that hexane fraction of Brazilian propolis had the highest antiadherent action relative to *Streptococcus mutans* and *Streptococcus sobrinus* [55]. 

Additionally, propolis turned out to lead to the decreasing of glucosyltransferase activity. The glucosyltransferases such as GTF, E.C. 2.4.1.5, are important agents that have impact on accumulation of a dental plaque. Water insoluble glucan facilitates adsorption of bacteria to teeth and creates a stable connection between streptococci and dental membrane. In this way, it increases the accumulation of a dental plaque and adherence of bacteria to teeth. Apigenin, a 4′,5,7-trihydroxyflavone, is an important component in composition of propolis being responsible for inhibiting *Streptococcus mutans* glucosyltransferase activity [56]. 

Besides apigenin, kaempherol and cinnamic acid possess the ability to inhibition of glucosyltransferase activity (Table 1). Apigenin and kaempferol exhibit these properties at concentrations in a range from 67.5% to 140.0 mg/mL and cinnamic acid at concentration in range from 5.0 to 7.5 mg/mL [56,57,58]. 

The research conducted by Koo et al. confirmed that 3% ethanol extract of propolis reduced index plaque about 44.7% and inhibited formation of insoluble polysaccharide about 61.7% [59]. 

As has been mentioned above, propolis possesses an anti-inflammatory activity, which brings advantages in the prevention of dental plaque and periodontitis. Bacteria and their products occurring in the biofilm are responsible for the activation of immunocompetent cells to produce and release inflammatory mediators that lead to the destruction of periodontal tissue and initiate inflammation. Therefore, a dental plaque, which leads to inflammation, causes a cytokine storm. Thus, the association between altered biofilm and cytokine storm is connected with complications among patients with COVID-19 [13]. 

Due to the presence of flavonoids in propolis, the latter exhibits anti-inflammatory capability. Galangin and CAPE are the main propolis components accountable for anti-inflammatory properties of that natural product (Table 1) [12]. 

**Figure 3:**
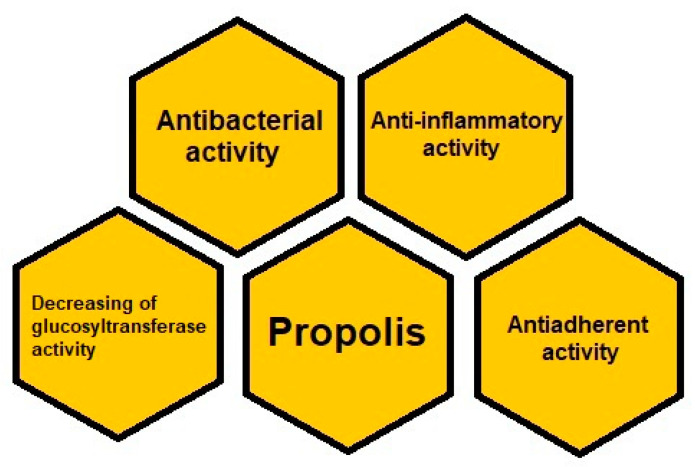
Anti-plaque action of propolis.

Therefore, the use of propolis in dentistry brings the potential therapeutic action (Figure 3). Due to propolis properties, the products containing that natural product are available worldwide and applied in the management of oral care. It has been shown that water with the addition of propolis ethanol extract as a mouthwash can reduce dental plaque. The beneficial effect in the reduction of dental plaque retention has been found while using the propolis containing toothpaste [10]. 

Machorowska-Pieniążek et al. confirmed the positive effect of propolis on children with cleft lip and palate treated with fixed orthodontic appliances. It is known, that malocclusion, teeth crowding combined with orthodontic appliances lead to an accumulation of dental plaque. In the mentioned study, toothpaste with 3% ethanol extract of Brazilian propolis was used among twenty-one subjects, three times a day. Control group included twenty subjects who used toothpaste without propolis, three times a day, as well. During 35 days, the examinations of oral hygiene and gingivitis were conducted among patients, using the lighting, mirror, probe, as well as bead probe. The assessment of propolis influence on dental plaque was evaluated using Approximate Plaque Index (API), Orthodontic Plaque Index (OPI), Gingival Index (GI), and supragingival bacterial plaque, which were taken at the beginning of the experiment and after 35 days. The results showed that ethanol extract of propolis decreased GI and OPI and the percentage of the *Actinomyces* spp. and *Capnocytophaga* spp. in propolis-treated group as compared with control group [60]. 

The results of the above-mentioned study show that propolis possesses antibacterial and anti-inflammatory properties and is useful in the reduction of a dental plaque especially during orthodontic treatment. 

It is worth mentioning that propolis is a natural and reliable antimicrobial alternative to chlorhexidine, which, despite its antibacterial properties, also has contraindications and cannot be used for a long period of time because of its typical staining of mucosa. 

### 3.3. The Role of a Diet in the Reduction of the Dental Plaque Development 

Natural components of food, especially polyphenols, influence the oral microflora, inhibiting the growth and metabolism of cariogenic bacteria. Polyphenols are abundant micronutrients in diet and their role in prevention of different diseases and dental plaque is well known. Polyphenols consist of a large group of chemical compounds found in food. These constituents are present in fruits, tea, coffee, cocoa, herbs, and spices. It was found that they take part in caries reduction. The mechanism of their anticariogenic action is based on the inhibition of cariogenic bacteria growth by inhibiting their metabolism [61]. Furthermore, inhibition of cariogenic bacteria growth lead to the reduction of bacterial acid production. Moreover, polyphenols inhibit salivary glucosyltransferase and amylase activity, and decrease the *Streptococcus mutans* adherence to a dental surface. Due to polyphenols, food products rich in these compounds have a beneficial impact on oral health [12]. 

Among fruits, the main role in the prevention of dental caries and dental plaque play: pomegranate fruits, raisins, and cranberries. 

Polyphenols included in pomegranate fruits such as ellagitannin, inhibit the adherence of cariogenic bacteria to teeth surface [62]. 

Raisins constitute a rich source of polyphenols, among which the most abundant ones are the flavonols like quercetin and kaempferol, and phenolic acids such as caftaric and coutaric acid. They all decrease pH value to 6 inside the oral cavity. Moreover, they inhibit the growth of oral pathogens. Due to the polyphenols occurrence in cranberries, they play an important role in anti-caries and anti-plaque effects. Cranberry constitutes a rich source of several classes of bioactive flavonoids including flavanols, anthocyanins, and proanthocyanidins. The polyphenolic fraction of cranberry juice inhibits the development of biofilm and inhibits the production of bacterial acids by cariogenic *Streptococci*. The cranberry fraction was found to deactivate the enzymes such as glucosyltransferase and fructosyltransferase, thus decreasing the adhesion of *Streptococci* to the tooth surface, followed by the inhibition of the plaque formation [63,64,65]. 

Polyphenols contained in green tea, such as gallusan epigallocatechin, gallusan epicatechin, epigallocatechin, and epicatechin cause the inhibition of dental caries development in rats, in about 40% [66]. 

Moreover, Awadella et al. indicated that rinsing the mouth with 2% infusion of green tea causes a reduction of *Streptococcus mutans* amount in dental plaque. Green tea decreases pH value, and by this way prevents the development of dental caries. In addition, rinsing mouth with 1.5% infusion of black tea brings advantages, as well. Therefore, drinking tea after a meal rich in carbohydrates inhibits the α-amylase activity and reduces the index of a plaque. Due to the α-amylase inhibition, the production of bacterial polysaccharides is limited [67]. 

On the other hand, coffee contains chlorogenic acid, which inhibits the glucosyltransferase activity and adsorption of *Streptococcus mutans* to the teeth. Glucosyltransferases take part in glucans synthesis, which leads to the adhesion of cariogenic bacteria to enamel surface and allows bacteria aggregation. [68,69]. 

In turn, polyphenols in cocoa cause the reduction of biofilm formation and acids production by *Streptococcus mutans* and *Streptococcus sanguis*. Moreover, cacao ingredients inhibit the bacterial glucosyltransferase activity [70]. 

Polyphenols occur in herbs and spices, as well. Important roles in oral health care is played cloves spice, cinnamon, nutmeg, ginger and licorice [71]. 

Clove spices contain a large amount of phenolic compounds and eugenol, due to they are used in dentistry and exhibit growth-inhibitory activity against *Streptococcus mutans* [72]. 

Cinnamon was found to influence in a positive way the dental plaque reduction. It contains cinnamaldehyde, eugenol, and cinnamyl alcohol, which are responsible for inhibiting the adhesion bacteria to teeth and for inhibiting water insoluble glucans synthesis. Cinnamaldehyde is the most active against the two oral pathogens: *Streptococcus mutans* and *Streptococcus sobrinus* among the main constituents of cinnamon. Eugenol and cinnamyl alcohol exhibit greater effective activity against *Steptococcus sobrinus* than to *Strepotococcus mutans*. Moreover, eugenol demonstrates a strong antibacterial effect against *Porphyromonas gingivalis*. It reduces the biofilm formation of *Porphyromonas gingivalis* and possesses an ability to reduce the existing biofilm. Eugenol inhibits the acid production and the synthesis of water-insoluble glucans by *Streptococcus mutans*. Other components of cinnamon such as: linalool or β-caryophyllene may inhibit biofilm formation [73,74,75,76].

Nutmeg contains macelignan, which has the ability of biofilm reduction as well as exerts an antibacterial effect against *Streptococcus mutans* and *Lactobaccilii* spp. [77]. 

Ginger is a well-known source of polyphenols like gingerol and shogaol, thanks to which exhibits antibacterial action. Park et al. showed antibacterial activity of 10-gingerol and 12-gingerol against *Prevotella intermedia*, *Porphyromonas gingivalis*, and *Porphyromonas endodontalis*, which take part in periodontal infections. They described antibacterial activity of ginger against *Streptococcus mutans* and *Lactobacillus acidophilus* [78,79].

Licorice possesses antibacterial activity against *Streptococcus mutans*. In low concentration of 16 µg/mL, it turned out to prevent dental caries [80].

We would like to underline the importance of association between dietary flavonoids consumption and reduction of dental plaque accumulation as a risk of many systemic diseases. The daily polyphenols consumption in European countries is different, being equal approximately from 863 ± 415 mg/day in Finnish adults [81], 1193 ± 510 mg/day in French adults [82], 820 ± 323 mg/day in Spanish people [83], to 1756.5 ± 695.8 mg/day in Polish people [84]. The total intake of polyphenols is recommended about 1 g/day [85]. Total intake of polyphenols depends on nutritional habits. Results of analysis showed that in easter societies, polyphenols intake reaches even to 2 g in a daily dose. Recommended polyphenols intake, allowing the functioning of the body is equal to 250–500 mg, but we should increase polyphenols consumption because they play a crucial role in the diseases prevention and according to the recent research polyphenols exert a beneficial impact on prevention of a dental plaque accumulation [86]. 

Besides fruits, herbs, and spices, a propolis is a rich source of polyphenols, which are responsible for antimicrobial and antiadherent action of the mentioned natural bee product. Moreover, it inhibits bacterial glucosyltransferase activity [11,87]. 

Propolis plays an important role in reduction of a dental plaque. It is available as a natural supplement and functional food. Propolis ethanol extract is consumed as a health aid, which reduces plaque accumulation and periodontal complications. 

## 4. Conclusions

Dental biofilm is associated with the development of systemic diseases such as: cardiovascular diseases, rheumatoid arthritis, diabetes, and respiratory diseases, especially severe complications due to SARS-CoV-2 infection, as well as Alzheimer’s disease. As presented above, there may be a strong connection between dental plaque and the severity course of SARS-CoV-2 infection, because dental plaque plays an important role in exchanging of bacteria between the mouth and the lungs. Therefore, propolis as a natural substance, useful in dentistry and oral health management, can bring advantages in maintaining oral health and in prophylaxis of systemic diseases. Propolis possesses antibacterial, anti-adherent, and anti-inflammatory properties as well as inhibits glucosyltransferase activity. Extract of propolis reduces cell adhesion and biofilm formation. In dentistry, it may be used as an active component of toothpaste, mouth rinse, and chewing gum. In addition, a diet rich in polyphenols prevents cariogenic bacteria and reduces the inflammatory process. Moreover, propolis administered orally as a supplement and component of functional food may decrease plaque accumulation and gingivitis. 

The improvement of oral health by using propolis as cariostatic agent and diet supplemented with polyphenols may reduce the risk of COVID-19 complications. Keeping oral health in good condition may decrease the severity course of SARS-CoV-2 and reduce the associated morbidity. This fact should be taken into consideration during the pandemic of COVID-19.

The analysis of the literature proves that there is a lack of consistency and standardization in currently applied treatment protocols, therefore objective comparisons of available clinical trials are very difficult. The main limitations of the conducted research are small cohorts of patients and a lack of validation studies. In order for the research results to be more reliable, long-term studies on a larger group of patients should be conducted. 

## Figures and Tables

**Table 1:** Summarization of the action of propolis active components and correlation with their influence on the oral cavity condition.

Active	Action	Literature
Apigenin Kaempherol Cinnamic acid	Inhibition of the Glucosyltransferase activity	[58,59]
Galangin Chrysin Pinobanksin Quercetin Naringenin Apigenin TT-farnesol Phenolic acids like caffeic acid, benzoic acid, cinnamic acid, gallic acid Artepillin C (3,5-diprenyl-*p*-coumaric acid) Catechins Ursolic acid Bacarin	Antibacterial action	[7,8,9,10,11]
Not identified	Antiadherent action	
Galangin Caffeic acid phenethyl ester (CAPE)	Anti-inflammatory action	[12]
